# Hereditary Ovarian Carcinoma: Cancer Pathogenesis Looking beyond *BRCA1* and *BRCA2*

**DOI:** 10.3390/cells11030539

**Published:** 2022-02-04

**Authors:** David Samuel, Alexandra Diaz-Barbe, Andre Pinto, Matthew Schlumbrecht, Sophia George

**Affiliations:** 1 Division of Gynecologic Oncology, Department of Obstetrics, Gynecology and Reproductive Sciences, Leonard M. Miller School of Medicine, University of Miami, Miami, FL 33136, USA; 2 Sylvester Comprehensive Cancer Center, Miami, FL 33136, USA; 3 Department of Political Science, University of Miami, Coral Gables, FL 33146, USA; 4 Department of Pathology, Leonard M. Miller School of Medicine, University of Miami, Miami, FL 33136, USA

**Keywords:** hereditary breast and ovarian cancer syndrome, ovarian cancer pathogenesis, carcinogenesis, serous tubal intraepithelial carcinoma, STIC, *BRCA1*, *BRCA2*, *PALB2*, *RAD51*, *ATM*, *BRIP1*, *MRE11*, *NBN*, *RAD50*

## Abstract

Besides *BRCA1* and *BRCA2*, several other inheritable mutations have been identified that increase ovarian cancer risk. Surgical excision of the fallopian tubes and ovaries reduces ovarian cancer risk, but for some non-*BRCA* hereditary ovarian cancer mutations the benefit of this intervention is unclear. The fallopian tubes of women with hereditary ovarian cancer mutations provide many insights into the early events of carcinogenesis and process of malignant transformation. Here we review cancer pathogenesis in hereditary cases of ovarian cancer, the occurrence of pre-invasive lesions and occult carcinoma in mutation carriers and their clinical management.

## 1. Introduction

Familial or hereditary ovarian cancer syndromes (HOC) encompass a number of tumor suppressor genes in which heritable mutations together account for 24% of epithelial ovarian cancer cases [1]. The last decade has seen major clinical advances in the treatment of ovarian cancer including the use of angiogenesis inhibitors [2] and poly-ADP ribose polymerase inhibitors [3,4,5,6] which have dramatically improved overall survival in this disease. Despite these improvements, ovarian carcinoma remains the most lethal gynecologic malignancy with more than 21,000 new cases and 13,000 deaths in the U.S. in 2020 [7]. Among these, hereditary cancer syndromes are a prime target for early detection and prevention. Current guidelines recommend germline genetic testing for all women with ovarian cancer, cascade testing of family members and risk-reducing surgery for those with actionable mutations [8,9]. In this review, we will summarize what is known about hereditary ovarian cancer pathogenesis in women with germline mutations in several DNA repair pathways. 

**Figure 1:**
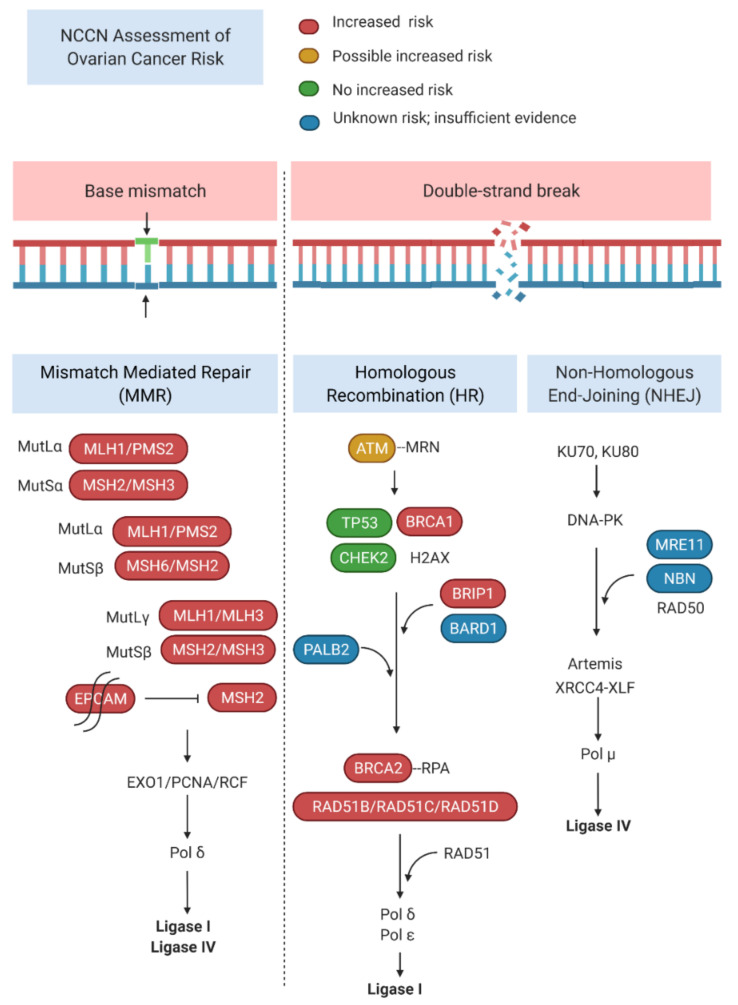
DNA repair pathways with genes associated with hereditary ovary cancer and their risk assessment according to the 2019 NCCN guidelines. Created with *Biorender*.

Multiple genes with pathogenic mutations in the germline have been implicated in hereditary ovarian cancer risk. The most commonly identified are *BRCA1* and *BRCA2* of the Fanconi anemia pathway, which are inherited in an autosomal dominant manner [10]. Loss-of-function mutation of a single allele confers a lifetime risk of ovarian cancer of 40% and 18% for *BRCA1* and *BRCA2*, respectively [11]. Mutations of other genes in the Fanconi anemia pathway also confer increased ovarian cancer risk, including *PALB2* [12,13]; *ATM* [14]; *RAD51C/D* [15,16,17]; and *BRIP1* [18,19]. Mutations of the mismatch DNA repair pathway genes *MLH1*, *MSH2*, and *MSH6* are associated with an increased lifetime risk of ovarian cancer (11% for *MLH1*, 17.4% for *MSH2*, and 10.8% for *MSH6* by age 75) [20]. (Figure 1). 

Current evidence indicates the fimbriae of the fallopian tube, rather than the ovary is the etiological site of high grade serous ovarian cancer (HGSC) in women with both sporadic and hereditary cases [21,22,23,24,25,26,27]. In 2001, Piek et al first reported dysplastic changes in the fallopian tube epithelia (FTE) of *BRCA* mutation carriers undergoing risk-reducing surgery [28]. These lesions, termed serous tubal intraepithelial carcinoma (STIC), the immediate precursor to HGSC arise from secretory cells, have p53 mutation with strong immunohistochemical staining and are highly proliferative [2,23,25,29,30,31]. Since these foundational developments, numerous studies have identified STIC as well as occult carcinoma in the fallopian tube [28,32,33,34,35]. 

The standard of care for women with *BRCA1* and *BRCA2* mutations is risk-reducing surgical resection of the fallopian tubes and ovaries (RRBSO) [8]. Mutations in non-*BRCA* HOC-associated genes are more frequently being detected with increased utilization of germline genetic screening but their optimal management is less clear. For several non-*BRCA* HOC mutations the National Comprehensive Cancer Network cites unknown risk or insufficient evidence to support increased screening or prophylaxis [8]. The mechanistic similarities and differences between carcinogenesis in *BRCA* mutation carriers, other HOC mutation carriers and sporadic cases remain unclear. Here, we discuss pre-neoplastic lesions and occult carcinoma occurring in women with *BRCA* mutations as well as other rare and recently described HOC mutations and describe the early events in carcinogenesis in this population. 

## 2. Pathogenesis in Hereditary Ovarian Cancer: Insights from Risk-Reducing Surgery Specimens 

The fallopian tubes of women with HOC mutations who have undergone RRBSO provide important insights into the process of malignant transformation. Many previous studies have made inferences about ovarian cancer pathogenesis from high grade serous carcinoma cases with concurrent precursor lesions. There is, however, a concern that rapidly expanding invasive carcinoma destroys precursor lesions and fundamentally alters the precancerous environment of the tube [36,37]. What then can we learn from the histopathological and molecular genetic analysis of the fallopian tubes of women with HOC mutations but without cancer?

The distal fallopian tube epithelia (FTE) of *BRCA* mutations carriers shows several transcriptional differences from tubal epithelia of unaffected women. Monoallelic *BRCA1* and *BRCA2* mutation carriers have significant modulation of genes associated with transcriptional regulation, cell cycle control and cell adhesion [38]. Multiple pathways in carcinogenesis are implicated including MAPK, adipokine, TGF-B and p53 signaling [39]. Notably, the differentially expressed genes in *BRCA* mutation carriers closely resemble serous carcinoma, rather than those of the low-risk (non-carrier) fallopian tube. Other studies have shown transcriptional changes in tubal epithelia of *BRCA* mutation carriers that indicate altered responses to inflammation and cellular stress. Compared to wild-type, tubal epithelia from *BRCA1* mutation carriers have increased expression of NAMPT, an enzyme involved in glucose metabolism and cellular stress as well as C/EBP-δ, a transcription factor implicated in inflammation, DNA damage response and tumorigenesis [39,40]. The DNA damage and repair markers P53 and γ-H2A also show high nuclear expression in the FTE and ovarian surface epithelium of *BRCA1*/*2* mutation carriers compared to non-mutation carriers [41]. The physiologic process of ovulation exposes the tubal fimbriae to reactive oxygen species and induces DNA double strand breaks [42] and *BRCA* mutation carriers appear to be more susceptible to these stresses. The cyclic hormonal context provided by the ovulatory cycle cannot be ignored [43,44]. 

*BRCA1* and *BRCA2* heterozygous fallopian tubes collected during the post-ovulatory luteal phase have a global gene expression profile similar to high grade serous carcinoma but distinct from follicular phase mutation carriers and both follicular and luteal phase controls [39]. *BRCA1*-mutated luteal phase fallopian tubes also demonstrate differential expression of more than 100 inflammation-related and NF-κB signaling genes compared to post-ovulatory non-mutation carriers which likely contributes to tumorigenesis [45]. Despite these observed transcriptional changes, most women with *BRCA* mutations will never develop ovarian cancer, complicating the identification of the specific transcriptional program that leads to neoplasia. One study has addressed this challenge by using *BRCA1* fallopian tubes with STIC (i.e., demonstrated proclivity to neoplasia) and performing microdissection and expression profiling of the histologically normal epithelial regions [46]. The authors compared gene expression in this histologically normal FTE from mutation carriers to non-mutation carriers. Notably, nearly one quarter of the highly differentially expressed genes were also differentially expressed in *BRCA1*-associated carcinomas. The preneoplastic signature present in *BRCA1* mutation carriers included modulation of EFEMP1, involved with cell-cell communication and angiogenesis regulation; E2F3, a cell cycle regulator implicated in malignant transformation; and CSPG5, a growth factor and activator of HER2/neu, all of which have been described in human carcinogenesis from multiple disease sites. 

Epigenetic mechanisms also contribute to hereditary carcinogenesis and several studies have addressed this using risk-reducing surgery specimens from *BRCA* mutation carriers without cancer. FTE from *BRCA* mutation carriers show increased promoter methylation of tumor suppressor genes compared with non-mutation carriers [47,48]. This increased methylation pattern does not correspond to preneoplastic foci and there is no difference in methylation between p53 signatures and adjacent histologically normal FTE [47]. Disruption of existing epigenetic control is also a relevant mechanism. Analysis of differentially methylated genes in prophylactically removed fallopian tubes from *BRCA* mutation carriers shows the gene *HOXC4* is highly expressed in the fimbriae which is a key trigger for activation-induced deaminase and induces epigenetic reprogramming [48]. Bartlett et al show overexpression of activation induced deaminase in cultured fimbrial cells modifies the methylation pattern to one that closely resembles HGSC and cellular differentiation. Yet another epigenetic mechanism is monoubiquitination of histone H2B which is performed by *BRCA1* and regulates gene expression, DNA damage response and chromatin segregation [49]. In non-mutation carriers, there is incremental loss of monoubiquitination of histone H2B from histologically normal fallopian tube, to STIC to HGSC, indicating this is an early and progressive epigenetic step in carcinogenesis [50]. Interestingly, germline *BRCA1* mutants do not show loss of monoubiquitination of histone H2B compared to wild type, including after sub-analysis of *BRCA1* mutations specifically involving the functional domain responsible for ubiquitination [49]. While these findings need to be validated using larger sample sizes, they suggest one distinct mechanism of carcinogenesis occurring in sporadic but not hereditary cases. 

These findings add to the growing evidence of haploinsufficiency as a carcinogenic mechanism in HOC. Contrary to the “two-hit hypothesis” which requires loss-of-function mutations in both alleles of a tumor suppressor gene for tumorigenesis, the studies highlighted above indicate multiple pathogenic tubal changes occur with monoallelic germline *BRCA* mutation. Increased cancer susceptibility in the fallopian tube appears to begin with loss of a single allele of *BRCA1* or *BRCA2*. There is parallel evidence that other, non-*BRCA* mutations may also model haploinsufficiency. Lee et al [51] showed both monoallelic and biallelic *PALB2* mutated breast tumors have no significant difference in homologous recombination deficiency scores. Tumors with monoallelic germline *PALB2* mutations are also more likely to have high risk frameshift mutations than somatic *PALB2*-null tumors [52]. Additionally, both preneoplastic FTE lesions and primary peritoneal carcinoma have been reported in *PALB2* heterozygote patients [53,54]. Taken together, loss of heterozygosity does not appear to be the rate-limiting step in HOC pathogenesis. 

## 3. Types of Preneoplastic Fallopian Tube Lesions and Significance

**Figure 2:**
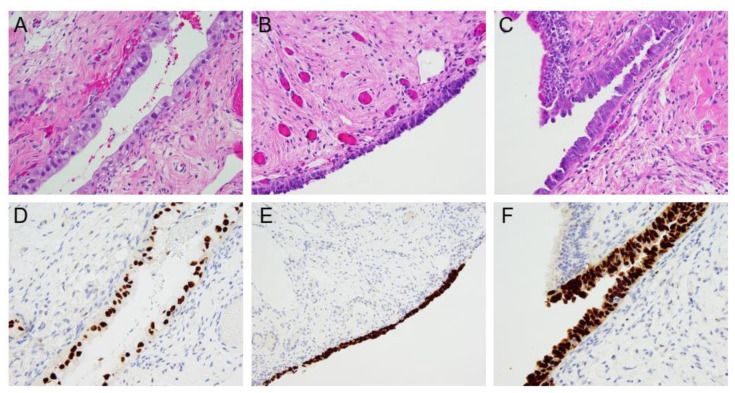
Histologic features of HGSC precursor lesions occurring in the fallopian tube. (**A**) p53 signature: hematoxylin and eosin (H&E) staining shows a single layer of cells, mostly non-ciliated, with abundant cytoplasm and bland cytologic features. (**B**) Serous tubal intraepithelial lesion (STIL): H&E shows non-stratified lining and lack of conspicuous nuclear atypia and is non-diagnostic for intraepithelial carcinoma. (**C**) Serous Tubal Intraepithelial Carcinoma (STIC): H&E shows the distal fallopian tube epithelium demonstrates absent cilia, loss of polarity and marked cytologic atypia. (**D**–**F**) Immunohistochemistry for p53 of the above sections shows aberrant staining and overexpression, a feature common to all tubal HGSC precursor lesions.

Several preneoplastic histopathological entities associated with HGSC have been identified in FTE including the p53-signature [30,31], secretory cell outgrowths (SCOUTs) [55], serous tubal intraepithelial lesions (STILs) and serous tubal intraepithelial carcinoma (STIC) [56] and are depicted in Figure 2. Secretory cell outgrowths (SCOUTs) are arrays of >30 cells found in either the proximal or distal tube with absent ciliated differentiation, loss of PAX2 and intact p53 [55]. It remains unclear if SCOUTs progress to other pre-neoplastic lesions or serous carcinoma. The p53 signature is a closely related entity but characterized by loss of p53 with accumulation of the deficient protein. The p53 signature occurs in the distal fallopian tube, involves an array of >12 benign secretory cells, exhibits strong p53 immunostaining, low proliferative index and co-localizes with gamma- H2AX [31,57]. In contrast, serous tubal intraepithelial carcinoma (STIC) is a noninvasive lesion characterized by nuclear enlargement and hyperchromasia, chromatin aggregation and strongly positive p53 immunostaining [58]. Additionally, they have loss of polarity, epithelial tufting, and high proliferative index [59]. Intermediate lesions with aberrant p53 expression but lacking these diagnostic features of STIC and low proliferation are termed serous tubal intraepithelial lesions (STIL) [60]. Although the clinical significance of some of these lesions remains unclear, there is growing evidence that they represent a spectrum of “early serous proliferations” that coexist with or precede the development of HGSC [61,62]. 

Evolutionary analyses show that p53 signatures and STICs are the precursors to ovarian carcinoma and ultimately metastases [63]. However, the paradigm of transformation of a single preneoplastic lesion into HGSC has been challenged by whole-exome mutational analysis of micro-dissected p53 signatures and STIC. Using this approach, Wu et al found multiple unique p53 mutations present in multiple STIC lesions isolated from a single patient. In patients with both STIC and occult carcinoma, phylogenetic analysis showed STIC was in some cases clonally related and in others, not [36]. This suggests multiple STIC may develop in tandem and divergently before one expands and becomes HGSC. 

## 4. Incidence of Preneoplastic Lesions and Occult Carcinoma in HOC Mutation Carriers

Reports on the incidence of STICs in patients with increased hereditary ovarian cancer risk vary widely [56]. The diagnosis of these rare lesions has poor inter/intra-observer reproducibility and is dependent on micro-sectioning technique [25,64]. For *BRCA1* and *BRCA2* mutation carriers, the incidence of isolated STIC was thought to be 3% based on pooled data from more than 2000 RRBSOs from nine studies [65]. Recent data indicate that the incidence is higher. A multicenter retrospective study of more than 450 *BRCA1*/2 mutation carriers undergoing RRBSO showed a 3.5% incidence of STIC but when a subset of these samples underwent detailed pathologic review, 12.4% were found to have either STIC, STIL or invasive carcinoma [66]. A prospective study which used the SEE-FIM protocol for all pathologic analyses found STIC lesions in 9.9% of 500 RRBSO specimens from patients with *BRCA1*/*2* mutations [67]. There is a paucity of clinical data available on the prevalence of precursor lesions in asymptomatic women with other, non-*BRCA* mutations implicated in HOC due to their rarity. The largest study of this population to date was performed by Rush et al and included 273 patients with non-*BRCA* HOC mutations or high-risk family history who underwent RRBSO. Isolated STIC was found in one *PALB2* mutation carrier and none were found in the high-risk family history group or among 41 patients with defects in other homologous recombination genes or mismatch repair genes [54]. One other study has identified intraepithelial neoplasia without invasive carcinoma in a *PALB2* mutation carrier, although only preliminary results are available [68]. A small retrospective study of 18 patients showed no serous intraepithelial precursor lesions among patients with *RAD51C*, *BARD1*, *BRIP1*, *PALB2* or *CHEK2* mutations who underwent RRBSO [11]. 

Multiple studies have reported on occult carcinoma detected at RRBSO, with estimates between 1–12% for *BRCA* mutations carriers [69]. A recent systematic review suggests the incidence may be at the lower end of published estimates [70]. Using 27 studies including over 6000 high risk women undergoing RRBSO, Piedimonte et al found a pooled prevalence of occult carcinoma of only 1.2%. This study was limited by excluding non-*BRCA* mutations and by combined analysis of *BRCA* mutations carriers with patients with high-risk family history, which could underestimate the true prevalence. A summary of the incidence of preneoplastic lesions and occult carcinoma identified following risk-reducing surgery including non-*BRCA* mutations is provided in Table 1. Though limited to a few studies with small sample sizes, these data can inform preoperative counselling and planning. They indicate there is minimal occult disease at time of risk-reducing surgery for most non-*BRCA* HOC mutations. Pathologic findings from larger cohorts of women with these rare mutations may support or refute the timing of risk reducing surgery in this population. The unique observation of STIC in patients with *PALB2* mutations warrants further investigation. 

## 5. Clinical Management of Isolated Pre-Invasive Lesions in Hereditary Ovarian Cancer Mutation Carriers 

The optimal clinical management of HOC mutation carriers with isolated pre-invasive FTE lesions detected following prophylactic surgery remains unknown. A systematic review of 14 studies showed that management practices vary widely: 36% of patients received subsequent hysterectomy, 21% received lymph node assessment and 14% received adjuvant chemotherapy. Among women with *BRCA* mutations with STIC identified after RRBSO, 7.5% of patients later developed primary peritoneal carcinoma [71]. Management is thus directed at risk reduction for and early detection of primary peritoneal carcinoma. Surveillance with physical exam, CA-125 and ultrasound and CT imaging can be considered but may be low yield given that carcinoma develops several years after RRBSO [71,72]. Limited evidence argues against comprehensive staging surgery for this group. In one systematic review, zero patients with *BRCA1* or *BRCA2* mutations and isolated STIC who subsequently underwent staging surgery had invasive cancer [72]. Similarly, Wethington et al reported on seven patients with *BRCA* mutations and isolated STICs who underwent staging and none had other disease [73]. Adjuvant chemotherapy may not be warranted in cases of isolated STIC but data on use and development of primary peritoneal carcinoma are lacking [72].

## 6. Incidence of Ovarian Carcinoma with Rare HOC Susceptibility Mutations

Pathogenic variants in *PALB2, ATM* and *BARD1* are recognized by the NCCN as involved in hereditary breast and ovarian cancer syndrome but have insufficient evidence to determine their ovarian cancer risk [74].

### 6.1. BARD1

Massive parallel sequencing studies have shown *BARD1* loss-of-function mutation occurs in <1% of women with ovarian cancer [1]. Among women with *BARD1* mutations one study appeared to show an increased ovarian cancer risk (OR 4.2 95% CI 1.4-12.5) compared to population estimates from large, publicly available exome sequencing datasets [10]. Other U.S. cohorts compared to the same controls have not found *BARD1* to be significantly associated with ovarian carcinoma [75]. 

### 6.2. PALB2

*PALB2* was initially reported to occur at similar frequency in ovarian cancer to controls in large European cohorts [76]. However, the largest study to-date of patients with *PALB2* mutations including more than 500 families found a relative risk of ovarian cancer of 2.91 (95% CI 1.40-6.04) compared to country-specific population incidence [77]. Primary peritoneal carcinoma has also been reported in a patient with *PALB2* mutation following RRBSO [53]. 

### 6.3. ATM

For *ATM*, the prevalence of this mutation was 0.6% out of more than 1500 women with ovarian cancer reported to SEER registries. *ATM* was associated with a relative risk between 2.25 and 2.97% and has been estimated from clinical trial populations [10] using Exome Aggregation Consortium controls [78] and local controls [79]. 

Recent evidence suggests *PALB2* and *ATM* are associated with increased risk and expanded screening and risk-reducing surgery should be investigated for these groups. Studies of *BARD1* mutation and ovarian cancer risk are contradictory and the link remains unclear. 

The MRN complex (*MRE11, RAD50*, and *NBN*) is implicated in nonhomologous end joining DNA repair and has rarely been reported in association with ovarian cancer.

### 6.4. NBN

One biallelic *NBN* germline mutation was identified from 354 Russian women with ovarian carcinoma [80]. Other reports from the same geographic region have identified the same founder mutation in 1.7% of women with ovarian cancer [81]. However, in another study comparing more than 3000 women with epithelial ovarian cancer to healthy controls, there was no difference in *NBN* mutation frequency [76]. 

### 6.5. MRE11

The first report of *MRE11*-associated ovarian carcinoma was from a cohort of 151 women with hereditary breast or ovarian cancer, of whom one was found to have a germline *MRE11* mutation [82]. Parallel genomic sequencing of 360 women with pelvic serous carcinoma also identified one patient with this mutation [1]. Conversely, this patient had a concurrent *BRCA2* mutation and tumor analysis showed loss of heterozygosity for *BRCA2* only. Another study did not find an increased incidence of MRE11 mutation in women with ovarian cancer [10]. 

### 6.6. RAD50

*RAD50* mutations were not identified in a prospective series of 62 patients with ovarian carcinoma [83] but were found in another unselected ovarian cancer cohort with one affected patient out of 360 [1]. 

The studies of MRN complex (RAD50, MRE11, NBN) mutations and ovarian cancer summarized above are contradictory and are insufficient to determine ovarian cancer risk. Further investigation of the impact of pathogenic variants in these genes is needed.

## 7. Strategies for Risk-Reduction in HOC Mutation Carriers

Ovarian cancer screening is ineffective for women of average risk but may have a role for those at high risk due to HOC mutations or family history. Screening with a combination of serial CA-125 and pelvic ultrasound is commonly used for women in this high-risk group. NCCN guidelines note this strategy can be considered but that it has not been shown to decrease ovarian cancer mortality [8,84]. Studies specific to high ovarian cancer risk populations have shown that screening with pelvic ultrasound and CA-125 results in diagnosis at earlier stage [85,86] and may improve survival [86], although an ovarian cancer-specific survival benefit has not been demonstrated. In the absence of data on screening for women with non-*BRCA* HOC mutations, it is reasonable to consider this approach for this group. 

Surgical risk-reduction with bilateral salpingo-oophorectomy is the mainstay of management for women with HOC mutations. In *BRCA1* and *BRCA2* mutation carriers, RRBSO reduces ovarian, fallopian tube and peritoneal cancer risk by 80% and all-cause mortality by 68% [87]. *BRCA1* mutation carriers are recommended to undergo a RRBSO between 35 and 40 years of age, while *BRCA2* mutation carriers may delay the procedure until age 40 and 45 because of later presentation of disease [88]. There are currently no prospective data available on RRBSO for other non-*BRCA* HOC mutation carriers. The mutation-specific lifetime risk of ovarian cancer compared to *BRCA*-negative/family history positive women (2.6%) has been used as a threshold to guide management [8,89]. On this basis, current guidelines recommend RRBSO or consideration of RRBSO for patients with *BRIP1, MSH2, MLH1, EPCAM, RAD51C* and *RAD51D* mutations [8] (Table 2). Family history and other independent risk factors for ovarian cancer should also be considered in timing RRBSO. However, there remain several genes associated with HOC but with insufficient evidence to determine their ovarian cancer risk or to recommend surgical prophylaxis (Figure 1).

While the indication for RRBSO is reduction of ovarian cancer risk, this intervention may have a secondary effect of reducing breast cancer risk. In a study of 876 women with *BRCA1* and *BRCA2* mutations, Choi et al found a decrease in breast cancer incidence in the first five years following RRBSO and a weaker but significant decrease in long-term risk for *BRCA1* mutation carriers only [90]. A study by Stjepanovic et al also found that premenopausal RRSBO decreased short term risk of breast cancer in *BRCA1* mutation carriers, though longer follow-up and larger sample size is needed to determine the potential benefit in *BRCA2* carriers [91]. For women with other HOC mutations associated with significant breast cancer risk such as *CHEK2, PALB2* and *ATM*, the impact of RRBSO on breast cancer risk remains unknown. 

Chemoprevention with oral contraceptives can reduce cancer risk for women with HOC who desire contraception but have not completed childbearing or undergone RRBSO. For women with *BRCA1* and *BRCA2* mutations, oral contraceptive use reduces the risk of ovarian cancer by 50% with a further 36% risk reduction for each additional 10 years of use [92]. There are mixed data [93] on the associated breast cancer risk for women with *BRCA* mutations receiving oral contraceptives with no significant risk in some reports [92,94] and mild to moderate increased risk in others [95,96]. Patients with HOC mutations should be informed of the possible increase in breast cancer risk when initiating oral contraceptives.

Two studies have shown aspirin may decrease ovarian cancer risk in the general population and this approach may prove useful for women with HOC mutations. A pooled analysis of the Ovarian Cancer Association Consortium found that aspirin use was modestly associated with reduced ovarian cancer risk (OR 0.91 95% CI 0.84-0.99) [97]. A Danish case-control study found no difference in risk for anytime aspirin users but continuous, long-term aspirin use was associated with decreased epithelial ovarian cancer risk (OR 0.56 95% CI 0.32-0.97) [98]. The randomized phase II STICs and Stones trial (NCT03480776) is currently underway to evaluate aspirin use in women with *BRCA1* and *BRCA2* mutations prior to RRBSO.

## 8. Ongoing Clinical Trials

Several clinical trials are currently exploring novel risk-reducing interventions for women with high-risk hereditary ovarian cancer mutations and are detailed in Table 3. Although effective in decreasing ovarian cancer risk, RRBSO has many negative sequelae including increased risk of stroke and cardiovascular disease, dementia, and osteoporosis [99]. Besides this, women undergoing oophorectomy are frequently premenopausal, with surgery prompting abrupt genitourinary syndrome of menopause, sexual dysfunction, vasomotor symptoms and mood disturbances which negatively impact quality of life [100]. Given the evidence that the fimbriae of the fallopian tube, rather than the ovary is the etiological site of high-grade serous carcinoma, RRBSO may not be the only preventative strategy, particularly for patients with HOC mutations that impart lower ovarian cancer risk. 

Two ongoing clinical trials investigate ovarian conservation in women with HOC mutations. *SOROCk* (NCT04251052) will investigate the efficacy of risk reduction following salpingectomy only and is limited to patients with *BRCA1* mutations. In the *Radical Fimbriectomy* trial (NCT01608074) patients with *BRCA1*/*2* mutations will undergo laparoscopic resection of the fallopian tube and fimbrio-ovarian junction with ovarian conservation. The preliminary data from this trial presented in 2018 demonstrated that of 121 patients who underwent this procedure, 1.7% had STIC and 0.8% had invasive carcinoma with an overall favorable safety profile [101]. 

Alternately, a staged procedure with salpingectomy followed by interval oophorectomy could temporarily preserve ovarian function while still offering risk reduction. *TUBA-WISP-II* (NCT04294927) seeks to address the safety and efficacy of this staged approach. *TUBA-WISP-II* will examine the primary outcome of high grade serous ovarian cancer incidence and secondary outcomes of peri-operative morbidity and completion of oophorectomy for women with *BRCA* and other HOC mutations. Both trials include women with *BRCA* and other HOC mutations. Lastly, the *PROTECTOR* (ISRCTN25173360) and *WISP* (NCT02760849) trials will focus on quality of life outcomes following staged RRBSO including sexual function, psychological well-being, and satisfaction/regret [102]. Preliminary data from *WISP* presented in 2019 showed no patients had developed carcinoma and women who underwent a staged procedure had fewer menopausal symptoms and less decision regret, even when compared to women who received hormone replacement therapy [68].

## 9. Conclusions

Ovarian cancer pathogenesis in hereditary cases begins with altered responses to inflammation and cellular stress in the fallopian tube which occur in the context of cyclic hormonal control and ovulation. Epigenetic reprogramming and loss of monoubiquitination of histone H2B also play roles in tumor initiation. Multiple pathogenic changes are observed in the tubal fimbria of women with *BRCA* and other monoallelic tumor suppressor gene mutations, indicating cancer susceptibility is not dependent on loss of heterozygosity. Numerous studies have focused on the cancer risks of *BRCA1* and *BRCA2*, but increased attention is needed to the multitude of non-*BRCA* mutations that impart ovarian cancer risk. So far, tubal preneoplastic lesions have been identified in *PALB2* mutation carriers but not in those with other homologous recombination or mismatch repair gene mutations. Use of germline genetic screening is expanding, and we can expect to see greater numbers of patients with these rare mutations prior to cancer diagnosis. Delineation of mutation-specific cancer risks and the benefits of preventative interventions will allow more personalized management of ovarian cancer risk that also minimizes harm. 

## Figures and Tables

**Table 1:** Incidence of isolated serous tubal intraepithelial carcinoma (STIC) or occult carcinoma (OC) in non-*BRCA* germline mutation carriers following risk-reducing surgery.

**Mutation**	**Incidence**	**Lesions**	**Reference**
*BARD1*	0/4 0/1	STIC or OC STIC or OC	53 11
*BRIP1*	0/9 0/2	STIC or OC STIC or OC	53 11
*PALB2*	1/10 1/? 0/3	STIC STIC STIC or OC	53 69 11
*MLH1*	0/1	STIC or OC	53
*MSH2*	0/4	STIC or OC	53
*MSH6*	0/8	STIC or OC	53
*PMS2*	0/2	STIC or OC	53
*RAD51C*	0/2 0/4	STIC or OC STIC or OC	53 11
*RAD51D*	0/2	STIC or OC	53

**Table 2:** Summary of HOC mutations with increased or unknown risk of ovarian carcinoma and recommended management.

**Gene**	**Ovarian Cancer Risk**	**Recommended Management**
*ATM*	Possible increased risk	Insufficient evidence for RRBSO
*BARD1*	Unknown risk or insufficient evidence	
*BRCA1*	Increased risk	RRBSO at 35–40 years or completion of childbearing
*BRCA2*	RRBSO at 40–45 years or completion of childbearing	Increased risk
*BRIP1*	Increased risk	Consider RRBSO at 45–50 years
*MSH2, MLH1, EPCAM*	Increased risk	Consider RRBSO, timing individualized
*MSH6, PMS2*	Increased risk	Insufficient evidence for RRBSO
*NBN*	Unknown risk or insufficient evidence	
*PALB2*	Unknown risk or insufficient evidence	
*RAD51C*	Increased risk	Consider RRBSO at 45–50 years
*RAD51D*	Increased risk	Consider RRBSO at 45–50 years
*STK11*	Increased risk of non-epithelial ovarian cancer	

Adapted from NCCN Clinical practice guidelines in oncology- Genetic/Familial High-Risk Assessment: Breast and Ovarian (January 2019).

**Table 3:** Ongoing clinical trials of risk reduction strategies for HOC. RRS = risk-reducing salpingectomy; RRO= risk reducing oophorectomy; RRBSO= concurrent risk-reducing salpingo-oophorectomy.

	**TUBA-WISP-II**	**SOROCk**	**Radical Fimbriectomy**	**PROTECTOR**	**WISP**	**STICS and STONEs**
Identifier	NCT04294927	NCT04251052	NCT01608074	ISRCTN25173360	NCT02760849	NCT03480776
Trial design	2-arm non randomized	2-arm non randomized	Single-arm	3-arm non randomized	2-arm non randomized	2-arm randomized
Number of patients	3000 (estimated)	2262 (estimated)	123 (actual)	1000 (estimated)	423 (actual)	414 (estimated)
Treatment arms	1. RRS + RRO 2. RRSO	1. RRS 2. RRSO	1. Bilateral radical fimbriectomy.	1. RRS + RRO 2. RRSO 3. No surgery	1. RRS + RRO 2. RRSO	1. Daily aspirin 2. Placebo
Patient population	Premenopausal women aged 25–50 years old with *BRCA1*, *BRCA2*, *RAD51C, RAD51D,* or *BRIP1* germline mutation.	Premenopausal women aged 35–50 years old with a pathogenic or likely pathogenic germline *BRCA1* mutation.	Premenopausal women over 35 with a *BRCA1*/2 mutation or family history of breast/ovarian cancer.	Premenopausal women with increased genetic risk due to genetic mutation (*BRCA1*, *BRCA2*, *RAD51C, RAD51D, BRIP1*) or strong family history.	Premenopausal women aged 30–50 years old with a deleterious mutation in: *BRCA1*, *BRCA2*, *BRIP1, PALB2, RAD51C, RAD51D, BARD1, MSH2, MSH6, MLH1, PMS2, or EPCAM.*	Adult women with *BRCA1*/*BRCA2* germline mutation planning risk-reducing surgery in 6 months to 2 years.
Primary outcome	High grade serous ovarian cancer incidence	Time to development of high grade serous carcinoma	Rate of pelvic cancer	Sexual function	Change in Female Sexual Function Index	Premalignant and malignant lesions found after RRBSO
Secondary outcomes	Incidence of pre-malignant findings in tubes/ovaries Perioperative mortality and morbidity Incidence of pelvic cancer Incidence of breast cancer Uptake of risk reducing oophorectomy	Health-related quality of life Sexual dysfunction Menopausal symptoms Cancer distress Estrogen deprivation symptoms Medical decision making Adverse events	Surgical morbidity Rate of occult lesions on fimbriectomy specimens Incidence of breast cancer and recurrence of breast cancer Rate of secondary oophorectomy and associated morbidity Proteomic profile of tissue from radical fimbriectomy	Endocrine function/menopause Quality of life Satisfaction/regret Surgical morbidity Psychological health Incidence of STIC and invasive carcinoma Utility score Cost effectiveness	Severity of menopausal symptoms Quality of life Mental health Completion of oophorectomy	Patient-reported acceptance of the intervention Compliance (serum monitoring)
Estimated primary completion date	February 2040	October 2032	December 2019	July 2028	May 2041	December 2023

## Data Availability

Not applicable.
